# Functional reorganisation and recovery following cortical lesions: A preliminary study in macaque monkeys

**DOI:** 10.1016/j.neuropsychologia.2018.08.024

**Published:** 2018-10

**Authors:** Matthew Ainsworth, Helen Browncross, Daniel J. Mitchell, Anna S. Mitchell, Richard E. Passingham, Mark J. Buckley, John Duncan, Andrew H. Bell

**Affiliations:** a MRC Cognition and Brain Sciences Unit, University of Cambridge, 15 Chaucer Road, Cambridge, UK; b Dept. of Experimental Psychology, University of Oxford, Parks Road, Oxford, UK

**Keywords:** Prefrontal cortex, Neuropsychology, Resting state covariance, FMRI, Plasticity, Recovery of function

## Abstract

Damage following traumatic brain injury or stroke can often extend beyond the boundaries of the initial insult and can lead to maladaptive cortical reorganisation. On the other hand, beneficial cortical reorganisation leading to recovery of function can also occur. We used resting state FMRI to investigate how cortical networks in the macaque brain change across time in response to lesions to the prefrontal cortex, and how this reorganisation correlated with changes in behavioural performance in cognitive tasks. After prelesion testing and scanning, two monkeys received a lesion to regions surrounding the left principal sulcus followed by periodic testing and scanning. Later, the animals received another lesion to the opposite hemisphere and additional testing and scanning. Following the first lesion, we observed both a behavioural impairment and decrease in functional connectivity, predominantly in frontal-frontal networks. Approximately 8 weeks later, performance and connectivity patterns both improved. Following the second lesion, we observed a further behavioural deficit and decrease in connectivity that showed little recovery. We discuss how different mechanisms including alternate behavioural strategies and reorganisation of specific prefrontal networks may have led to improvements in behaviour. Further work will be needed to confirm these mechanisms.

## Highlights

• Lesions to prefrontal cortex result in decreases in functional connectivity.
• Decrease in functional connectivity associated with behavioural impairments.
• After 8 weeks, functional connectivity patterns recover along with behaviour.

Cortical damage that accompanies traumatic brain injury or stroke often extends beyond the boundaries of the initial injury. This can lead to maladaptive cortical reorganisation and cognitive impairment (Grefkes and Fink, 2014). On the other hand, beneficial cortical reorganisation following injury can also occur and this can lead to recovery of function. Understanding the nature of cortical reorganisation after injury and how this might be promoted is a challenge for research on developing treatments for patients suffering from brain injury.

Resting state functional magnetic resonance imaging (rsFMRI) provides an indirect method of measuring cortical organisation across the whole brain by correlating BOLD activation patterns between pairs of brain areas. Strong correlation implies, at minimum, a “functional” connection, and often an anatomical connection (Deco et al., 2011). Over the past decade, rsFMRI has been used to examine changes in network organisation in healthy individuals as well as patients who have suffered lesions or who have a variety of disorders such as schizophrenia, Alzheimer's and Parkinson's disease (Fornito et al., 2015, He et al., 2007, Siegel et al., 2016, Siegel et al., 2018). However, to fully understand the consequences of cortical reorganisation following damage, it is necessary to measure correlations within cortical networks both pre- and post-injury. It is virtually impossible to obtain pre-injury data from healthy human participants, while in patients, presurgical imaging does not reflect the status of a healthy brain. As such, we employ animal models, where we can collect data both before and after a lesion.

Recent studies have looked at functional connectivity following lesions in non-human primates. O'Reilly et al. (2013) sectioned the corpus callosum (with/without anterior commissure section) in monkeys to explore the relationship between structural connectivity and functional connectivity in neocortical areas. Grayson et al. (2016) used designer receptors exclusively activated by designer drugs (a.k.a. DREADDS) to temporarily inactivate the amygdala and were able to show how acute changes in functional connectivity in amygdala-cortical and cortico-cortical networks followed structural connectivity patterns. However, neither study related these changes to behaviour. By contrast, Meng et al. (2016) made neurotoxic lesions in the hippocampi of infant monkeys and correlated the resulting long-term changes in functional connectivity with performance on memory tests when the animals were 8–10 years old.

These studies are important in demonstrating the utility of studying functional connectivity following lesions in animals. However, to understand how behavioural recovery occurs after brain injury in patients, we need to correlate changes in functional connectivity with behavioural measures as recovery occurs. In the present study, we used rsFMRI to study how cortico-cortical connectivity in the macaque monkey brain changed in response to discrete lesions of the prefrontal cortex over an extended period of time; and how these changes related to behaviour. Specifically, we examined lesions of the cortex in upper and lower banks of the principal sulcus, which we refer to from here onwards as “PS-lesions”.

We chose to focus on PS lesions for several reasons. First, it is well known that lesions there reliably abolish the ability of monkeys to perform delayed response and delayed alternation tasks (Funahashi et al., 1993a, Miller and Cohen, 2001, Passingham and Wise, 2012). Tasks such as these are frequently used to probe working memory, a common impairment suffered by patients with damage to prefrontal cortex.

Second, numerous electrophysiological studies have examined the function of neurons near/within the principal sulcus (e.g., Funahashi et al., 1989; Funahashi et al., 1990, Funahashi et al., 1991; Funahashi et al., 1993a, 1993b; Kojima and Goldman-Rakic, 1984; Lebedev et al., 2004; Meyer et al., 2011) and our understanding of the characteristics of these neurons and their role in working memory is relatively sophisticated.

Finally, these areas are known to have extensive anatomical connections to other frontal regions as well as with parietal and temporal regions (Petrides and Pandya, 1984, Saleem et al., 2014, Yeterian et al., 2012). In short, the advantage of studying this system is that it is well characterised, both in terms of its connections and in terms of its physiology and function. It is therefore well-suited to our purpose, which is to examine how an injury to a single region within a network affects connectivity across the network, and the subsequent consequences to performance in a well-established behavioural paradigm.

We trained two monkeys on a location-based and object-based delayed match-to-sample task. We collected rsFMRI data and behavioural data at periodic intervals during the prelesion period. The animals then first received a lesion to both banks of the left PS, including areas 46 and 9/46. Following a post-operative recovery period, we resumed periodic testing and scanning sessions. Later, they received a second lesion to the same region in the opposite hemisphere and they were once again tested and scanned at regular intervals.

## 1 Materials and methods

All animal procedures were conducted in accordance with the United Kingdom Animals (Scientific Procedures) Act (1986) and approved by the University of Oxford local ethical review panel and the UK Home Office Animal Inspectorate. All husbandry and welfare conditions complied with the guidelines of the European Directive (2010/63/EU) for the care and use of laboratory animals. Two adult male monkeys (*Macaca mulatta*, 8–11 kg), purpose-bred in the United Kingdom, were used in this study. The monkeys were pair-housed with varying forms of environmental enrichment, free access to water, and a 12-h light/dark cycle. Veterinary staff performed regular health and welfare assessments, which included formalized behavioural monitoring.

### 1.1 Overview

**Fig. 1:**
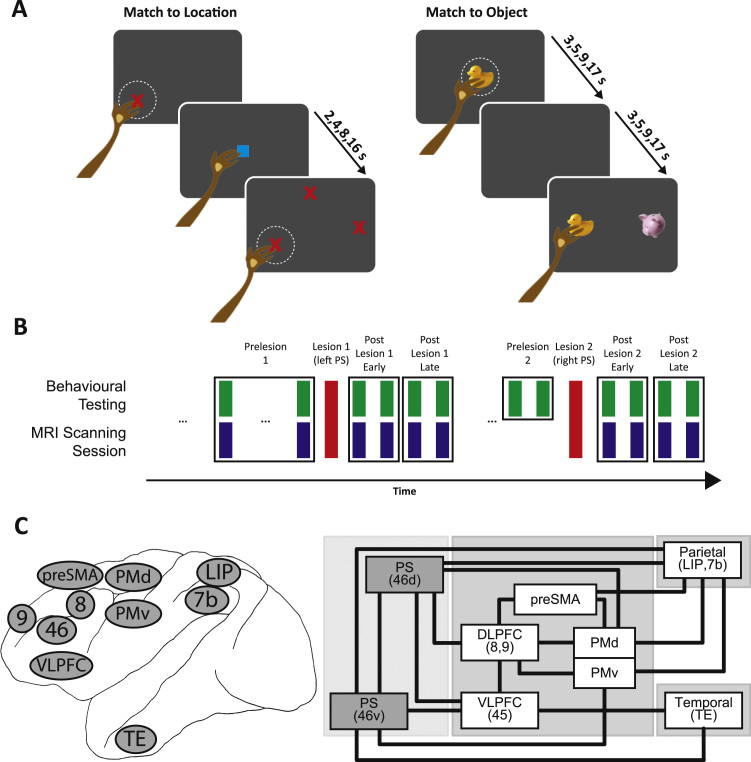
**Assessing behavioural impairments and cortical reorganisation following lesions to the principal sulcus.** A) Behavioural testing took place with the monkeys unrestrained in transport boxes, facing a touchscreen. In the ‘match-to-location’ task (left), the monkey was required to touch a cue that appeared in a random location on the touchscreen. The cue then disappeared and after a distractor (central blue square) and variable delay, three stimuli identical to the cue appeared in three different locations. The three locations included the sample location from the current trial, the cued location from a previous trial, and a third random location. The monkey was required to touch the location of the cue on the current trial to receive a food pellet reward. In the ‘match-to-object’ task (right), the monkey was once again required to touch a cue that appeared in a random location on the touchscreen. After a variable delay, two different stimuli appeared in random locations: the sample stimulus and a distracter stimulus. The monkey was required to touch the sample stimulus to receive a food pellet reward. B) Time course of our longitudinal experiment. We collected behavioural (green boxes) and MR (blue boxes) data at periodic intervals (approximately every 3–4 weeks) both before and after the PS lesions (red boxes). These sessions were grouped in pre- and post-lesion periods (indicated by square outlines). In the case of first prelesion period (Prelesion 1), the two scanning/behavioural sessions were separated by approximately 6 months. After the first lesion, scanning/behavioural data were collected every 3–4 weeks. In the case of the second prelesion period (Prelesion 2), we collected behavioural data at two timepoints prior to the lesion, separated by about 3–4 weeks. However, we were unable to collect MR data immediately prior to the second lesion, and so we used the MR data from the “Post-Lesion 1-Late” period as the prelesion data for the second lesion. C) Location and anatomical connectivity for regions in our network of interest. Connectivity based on Yeterian et al. (2012) and Saleem et al. (2014). TE: area TE; PMd: dorsal premotor area; PMv: ventral premotor area; LIP: lateral intraparietal area; 7b, 8, 9, 46: areas 7b, 8, 9, 46; VLPFC: ventrolateral prefrontal cortex; preSMA: pre-supplementary motor area; PS: principal sulcus.

**Fig. 2:**
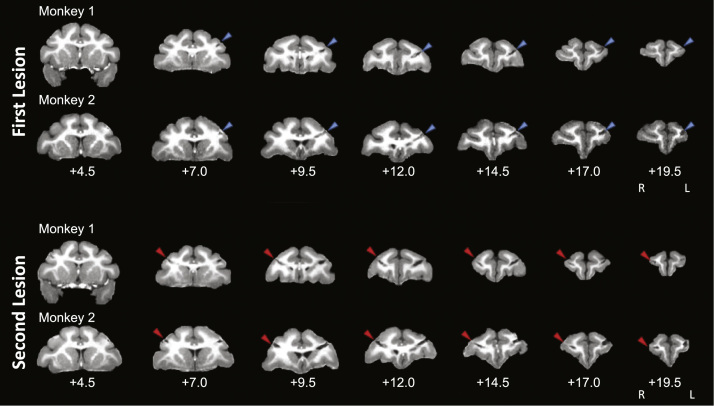
**Structural MRIs showing location of first and second lesions in monkey 1 and 2.** Coronal slices showing lesion to principal sulcus. Anatomical distances shown relative to the interaural axis.

We performed a longitudinal assessment of the effect of lesions to both banks of the principal sulcus (PS) on behavioural performance on two cognitive tasks and related it to changes in functional connectivity (Fig. 1A). Once the animals had reached a predefined level of performance on the behavioural tasks (> 70%), we collected resting state functional magnetic resonance imaging (rsFMRI) data under general anaesthesia at two intervals prior to the first lesion (Fig. 1B). Data from two additional scans, earlier in the animals’ training, are not included in the present report. Several days prior to each scanning session, the animals were tested on both location- and object-based delayed match-to-sample (DMS) tasks (see below). Following these two cycles of behavioural testing and scanning, each animal received a lesion to both the dorsal and ventral banks of the left principal sulcus (PS), targeting areas 46 and 9/46 (Fig. 2). Following a post-operative recovery period (approximately 4 weeks), we resumed cycles of behavioural testing and scanning (4 cycles approximately once/3–4 weeks). For the next several months, similar cycles of behavioural testing (but without scanning) continued. After 7 months following the first lesion, the animals received a second lesion to both banks of the right PS (Fig. 2). Following a post-operative recovery period (approximately 4 weeks), the animals were once again tested and scanned (4 cycles, approximately once every 3–4 weeks).

### 1.2 Behavioural tasks

Behavioural testing took place with the monkeys unrestrained inside small transport boxes (approximately 1 m^3^) (see Mitchell et al., 2007 for details). One side of the testing box faced a touchscreen to which the monkey had access. In the ‘match-to-location’ task (Fig. 1A, left), the monkey was required to touch a red cross that appeared in a random location on the touchscreen. The cross then disappeared and a distractor (blue square) appeared in the centre of the screen and the monkey was required to touch this. After a variable delay (2, 4, 8, or 16 s), three stimuli identical to the sample appeared in three different locations. The three locations included the sample location from the current trial, the sample location from a previous trial, and a third random location. The monkey was required to touch the cued location on the current trial to receive a food pellet reward.

In the ‘match-to-object’ task (Fig. 1A, right), the monkey was required to touch a cue that appeared in the centre of the touchscreen. There was then a variable delay (3, 5, 9, or 17 s; the extra 1 s was added to approximately match the distractor plus delay durations in the match-to-location task). Two stimuli then appeared on the touchscreen on either side of midline (along the horizontal meridian, equidistant from centre). These included the sample stimulus and a distracter stimulus (randomly allocated to either left or right of midline). The monkey was required to touch the stimulus that had been cued to receive a food pellet reward.

**Fig. 3:**
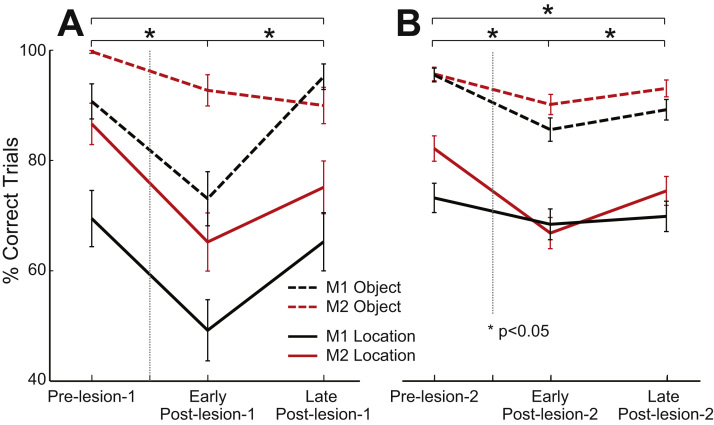
**Behavioural performance of both monkeys in the match-to-location (solid lines) and match-to-object (dashed lines) tasks.** Data are plotted as mean+/-SEM. A) Following a lesion to the left principal sulcus, both monkeys showed a significant drop in performance in both tasks in the testing sessions conducted within 8 weeks of the lesion. In the behavioural testing sessions beginning about 8 weeks post-lesion, behaviour showed overall improvement. B) After a second lesion to the right principal sulcus, there was again behavioural decline followed by partial improvement. (*p < 0.05).

The two tasks were not matched for overall difficulty: based on performance data, the location task was more difficult than the object task (Fig. 3). For each testing cycle, there were 1 or 2 test sessions per task (100–120 trials per session), on different days. The second test session was added from the second post-lesion 1 cycle onwards. For cycles with 2 test sessions per task, data from the two were combined as there was no significant difference in performance between the sessions when summed across all tasks, monkeys, and stages of testing (testing session 1 vs. testing session 2: 82 ± 2 vs. 83 ± 2%, p = 0.11, paired *t*-test).

Because of the relatively long delays between testing cycles (several weeks), we began each cycle with shorter ‘warm-up’ sessions (40–100 trials). These were held over two days prior to actual testing sessions in order to re-introduce the animals to the process of testing. Data obtained during warm-up days were excluded from all analyses.

All performance data (percent correct) were first arcsine transformed (to control for ceiling effects) (Studebaker, 1985) before being analysed using two separate three-way repeated-measures ANOVAs (one per lesion) with each testing cycle corresponding to a unit of replication. The three factors were task (match-to-location, match-to-object), monkey (monkey 1, 2), and experimental stage (pre-lesion-1/2, early post-lesion-1/2, late post-lesion-1/2). We examined all main effects, and the interaction between session and task. Post-hoc Tukey's HSD tests were carried out to identify changes in performance associated with experimental stage. Performance values are expressed as percent correct for data presentation and reporting of summary statistics.

### 1.3 Neuroimaging data collection

All imaging data were collected under general anaesthesia on a 3T scanner using a custom-made 4-channel phased array coil (H. Kolster, MRI Coil Laboratory, Laboratory voor Neuro en Psychofysiologie, KU Leuven). Procedures for inducing and maintaining general anaesthesia and the positioning of the monkeys in the scanner are similar to those described previously (Mars et al., 2011; Mitchell et al., 2016).

It is well-established that anaesthetic agent and level of anaesthesia can both have a significant effect on resting state data. For example, Hutchison et al. (2014) recently demonstrated that while doses of isoflurane between 1.0% and 1.5% reveal stable patterns of functional connectivity, doses higher than 1.5% result in dose-dependent decreases in overall connectivity – particularly between hemispheres. We therefore sought to maintain our level of anaesthesia within this recommended range. However, because each animal is different, and the physiology of the animal can differ from session to session, it is not possible to precisely control anaesthesia level from session to session without potentially compromising the welfare of the animal. We nonetheless made every effort to keep anaesthesia levels consistent from session to session in order to maximize consistency in the data. Animals were artificially respirated at a fixed rate, with a targeted anaesthesia level of approximately 1.5% inspired isoflurane. The actual range of anaesthesia levels over the entire project was ~ 0.9–2.2%, with a mean 1.52 ± 0.3% and mode of 1.5%.

Resting-state echo-planar images were collected at 2 × 2 × 2 mm resolution (36 axial slices, TR = 2 s, TE = 19 ms). We collected between 825 and 1600 volumes per scan session (mean: 1511, SD: 223), for an approximate scan duration of 56 min.

T1-weighted, high-resolution (0.5 mm isotropic voxels) structural images were collected using an MPRAGE (magnetization prepared gradient echo; TR = 2.5 s, TE = 4.01 ms, 3–5 averages) sequence. Anatomical images were corrected for coil inhomogeneity by dividing the MPRAGE data by a lower resolution MPRAGE sequence (1 ×1 ×1 mm) that did not include an inversion recovery pulse (Parameters: TR = 2.5 s, TE = 3.48 ms) as per the methodology outlined by Van de Moortele et al. (2009).

### 1.4 Neurosurgery

Lesions to the PS (areas 46 and 9/46) were performed under aseptic conditions using an operating microscope (for a detailed description of the surgical procedures, see Buckley et al., 2009; Buckley and Mitchell, 2016). To protect against intraoperative oedema and postoperative inflammation, steroids (methylprednisolone, 20 mg/kg, im) were administered the night before and three additional times 4–6 h apart (iv or im) on the day of surgery. Anaesthesia was initiated with a single dose of ketamine (10 mg/kg) and xylazine (0.25–0.5 mg/kg, im) and maintained using sevoflurane (min 1.0–2.0% to effect, in 100% oxygen) while the animal was mechanically ventilated. The animal was given atropine (0.05 mg/kg) to reduce secretions, an antibiotic (amoxicillin, 8.75 mg/kg) as prophylaxis against infection, and buprenorphine (0.01 mg/kg iv, repeated two times at 4- to 6-h intervals on the day of surgery iv or im) and meloxicam (0.2 mg/kg iv) for analgesia. Ranitidine, an H_2_ receptor antagonist (1 mg/kg iv), was given to protect against gastric ulceration as a side effect of the combination of steroid and nonsteroidal anti-inflammatory treatment. Heart rate, oxygen saturation, mean arterial blood pressure, end tidal CO_2_, body temperature, and respiration rate were monitored continuously throughout the procedure.

Under deep anaesthesia, the head was placed in a head holder and the skull exposed by opening the scalp and galea in layers. The temporal muscles were retracted, and a bone flap was removed. The dura was cut to expose the cortical surface. Both banks of the PS were removed with aspiration (Fig. 2). The dura was then sewn, and the bone flap replaced.

Following the procedure, the animals were monitored continuously for 48 h. Postoperative medication continued in consultation with veterinary staff. This included nonsteroidal anti-inflammatory analgesic (meloxicam, 0.2 mg/kg, oral) and antibiotic (8.75 mg/kg, oral) treatment following surgery in consultation with veterinary staff, typically for 5 days. In addition, steroids (dexamethasone, 1 mg/kg, im), once every 12 h for 3 days, then once every 24 h for 2 days; analgesia (buprenorphine, 0.01 mg/kg, im) for 48 h; and continued antibiotic treatment (amoxicillin, 8.75 mg/kg, oral) were also administered for 5 days. Gastric ulcer protection (omeprazole, 5 mg/kg, oral and antepsin, 500 mg/kg, oral) commenced 2 days prior to surgery and continued postoperatively for the duration of other prescribed medications, up to 5 days.

### 1.5 Data preprocessing and analysis

All data preprocessing and analysis was conducted using a combination of Matlab (The MathWorks Inc.), SPM8 (Statistical Parametric Mapping; www.fil.ion.ucl.ac.uk/spm), FSL (fMRI of the Brain (FMRIB) Software Library; http://fsl.fmrib.ox.ac.uk/fsl/fslwiki/; Jenkinson et al. (2012), Caret (Computerized Anatomical Reconstruction Toolkit; Van Essen (2012) and aa software (automatic analysis; Cusack et al. 2014; www.automaticanalysis.org). The methods used to analyse rsFMRI data are described in detail in Mitchell et al. (2016). Briefly, the structural volumes for each animal were aligned to standard space (112 Rhesus macaque template) – in the space of the atlas of Saleem and Logothetis (2006) using affine and nonlinear registration (SPM8) and segmented into grey matter, white matter and cerebrospinal fluid (CSF) tissue classes (McLaren et al., 2009). After removing the first 6 functional volumes, the remaining volumes from each rsFMRI dataset were used to estimate motion parameters (included as covariates of no-interest) and were aligned to standard space through a two-stage process. The data were spatially-smoothed with a 3 mm Gaussian kernel (full-width half maximum). Grey-matter masks were defined as voxels with grey-matter probability > 0.5 within each subject. Masks representing white matter, CSF, and the superior sagittal sinus were also produced and used to create covariates of no interest in time series analysis (see following).

Physiological covariates of no interest were constructed from the EPI time-series for white-matter and CSF tissue masks (up to 6 principal components each) (Behzadi et al., 2007). A further vascular component was defined as the mean time-course within a mask drawn for the superior sagittal sinus. The first temporal derivatives of these time-courses were also included. A motion confound covariate was defined as the time-course of average displacement over the expected brain volume (approximated as a sphere of radius 40 mm). The first temporal derivative of this vector was also included, as were the element-wise squares of both vectors. Discrete cosine transform covariates were added to implement a temporal band-pass filter (0.0025–0.05 Hz) as commonly used (Mantini et al., 2011, Vincent et al., 2007). After projecting covariates from each grey-matter time-series, functional connectivity (“connection strength”) was estimated as correlations between the mean grey-matter time-series, for a range of pre-defined regions of interest (ROIs).

### 1.6 Defining the network of interest

To evaluate changes in connectivity following the lesions, we compared pre- and post-lesion connectivity in a predefined “network of interest” (shown in Fig. 1C) composed of a subset of frontal, parietal, and temporal regions. These regions were selected on the basis of their anatomical connectivity to areas 46 and 9/46 (Saleem et al., 2014, Yeterian et al., 2012), specifically focusing on those that have been implicated in the performance of location- and object-based cognitive tasks (Passingham and Wise, 2012). These regions were delineated according the macaque cortical parcellation scheme proposed by Van Essen et al. (2012). They included in the frontal cortex: dorsal area 6DR (“PMd”), ventral area 6Val (“PMv”), medial area 6M (“preSMA”), areas 8Ac and 9 (areas 8 and 9, collectively referred to as “DLPFC”), area 45 (“VLPFC”). They included in the parietal cortex: area 7b, dorso- and ventro- lateral intraparietal areas (LIPd, and LIPv). Finally, they included in the temporal cortex: TE1–3d (“TE”). A schematic of the intrahemispheric anatomical connectivity between these regions and areas 46 and 9/46 is shown in Fig. 1C based on (Saleem et al., 2014, Yeterian et al., 2012).

### 1.7 Analysing changes in functional connectivity

To analyse changes in connectivity, we averaged the different pairwise connections between regions in the network of interest, according to hemisphere and lobe. This yielded 11 different values per subject and stage of the experiment, corresponding to all possible pairwise combinations of hemisphere and lobe [e.g., left-frontal to left-frontal (LF-LF), left-frontal to right-frontal (LF-RF), left-frontal to left-parietal (LF-LP), etc.]. Note that LF-RF and RF-LF yield identical values and so only a single group is used to summarise these data. The data were modelled in two separate ANOVAs (one per lesion). These ANOVAs also included the following factors: connection (11 levels), experimental stage (3 levels, pre-lesion-1/2, early post-lesion-1/2, late post-lesion-1/2), and monkey (2 levels). Each scanning session served as the replication. Post-hoc Tukey's HSD tests were carried out on ANOVA-derived estimated marginal means.

To assess the spatial extent of lesion-related changes in connectivity, we calculated two control ANOVAs from the averaged connectivity between cortical areas outside our network of interest. These regions included 81 ROIs (per hemisphere) in the parietal (33 ROIs), occipital (7 ROIs), and temporal (41 ROIs) lobes as defined by the macaque cortical parcellation scheme proposed by Van Essen et al. (2012). As before, we averaged the pairwise correlation coefficients according to lobes and hemisphere [e.g., left-parietal to left- parietal (LP-LP), left- parietal to right- parietal (LP-RP), left-parietal to left-temporal (LP-LT), etc.].

## 2 Results

### 2.1 Changes in behaviour and functional connectivity following unilateral lesion

The ability of the monkeys to perform the two DMS tasks was significantly impaired following a unilateral lesion of the left PS (Fig. 3A). We compared behavioural performance (measured as % correct) in the two sessions prior to the lesion (pre-lesion-1) with the first two sessions following the lesion (early post-lesion-1) and the two after 8 weeks (late post-lesion-1) using an ANOVA (Fig. 3A; see MATERIALS AND METHODS). Critically, a significant main effect of experimental stage was observed (*F*_(2,12)_ = 8.71, p = 0.0046). We also observed a significant main effect of task (*F*_(1,12)_ = 64.81, p < 0.0001) and of monkey (*F*_(1,12)_ = 18.32, p = 0.0011). We did not observe any significant interaction between session and task (*F*_(2,12)_ = 0.17, p = 0.85). Post-hoc comparisons revealed a significant difference between the pre-lesion and early post-lesion periods (p = 0.0039) and early- vs. late-post lesion periods (p = 0.0447), and no significant difference between pre-lesion and late post-lesion (p = 0.39).

**Fig. 4:**
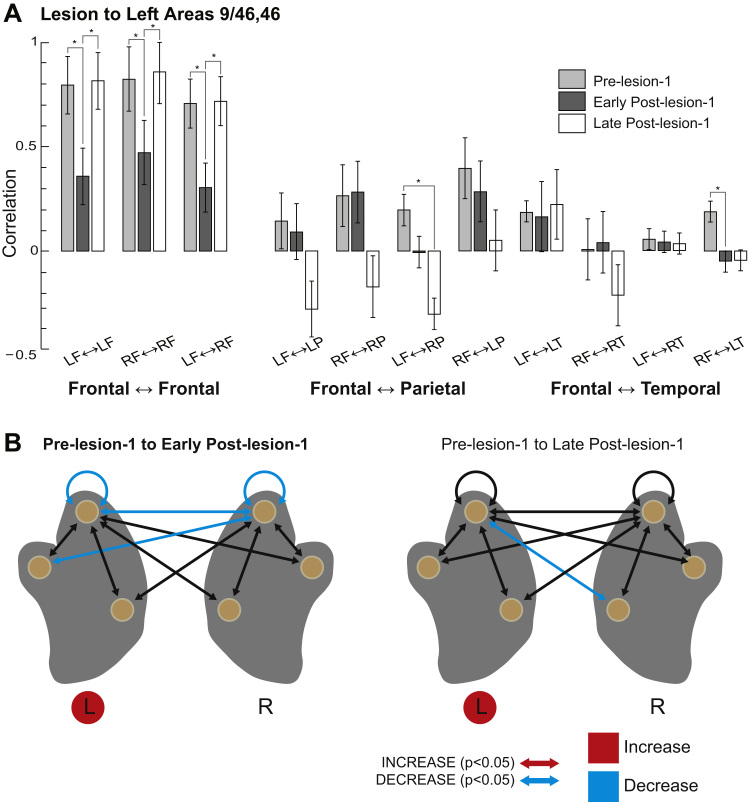
**Connectivity within frontal lobes impaired following lesion to regions near the left principal sulcus.** A) Average correlation for all frontal-frontal, frontal-parietal, and frontal-temporal connections in the pre-lesion, early-post-lesion (4–8 weeks post lesion) and late post-lesion (8–16 weeks) periods. B) Schematic representations of changes to connectivity in the early post-lesion (left) and late post-lesion (right) periods, relative to the pre-lesion period. Significant increases in functional connection strength (correlation) shown in red; decreases in blue. Filled red circles indicate lesioned hemispheres.

This decrease in behavioural performance during the first 8 weeks following the lesion (early post-lesion-1) was associated with significant changes in functional connectivity within our network of interest. In Fig. 4, we compare the changes in functional connectivity in the post-lesion periods to the pre-lesion levels, with the data grouped according to lobe and hemisphere. We analysed these data using an ANOVA (see MATERIALS AND METHODS). Matrices showing pairwise comparisons for the entire brain are shown in Supplementary Fig. 1.

We observed significant main effects for experimental stage (*F*_(2,6)_ = 12.19, p = 0.037) and connection (*F*_(10,60)_ = 28.62, p = 1.96 × 10^−6^), and a significant interaction between the two (*F*_(20,60)_ = 3.68, p = 0.020). We did not observe a main effect of monkey (*F*_(1,6)_ = 1.27, p = 0.302). A similar analysis looking at brain areas outside the network of interest (see MATERIALS AND METHODS) revealed no significant effects of experimental stage (*F*_(2,6)_ = 0.29, p = 0.76) or monkey (*F*_(1,6)_ = 2.13, p = 0.302); and no significant interaction between stage and connection (*F*_(40,120)_ = 0.92, p = 0.61) (see Supplementary Fig. 1A).

The most striking change in the first series of scans following the first lesion to the left PS was an overall decrease in correlation strength both within and between regions in the left and right frontal lobes (Fig. 4). The correlations between frontal and parietal areas were unchanged (p's > 0.05). The correlations between frontal and temporal areas were also unchanged, except for the average connection between right frontal and temporal lobes (p = 0.031; all the other frontal-temporal correlations p's > 0.05).

Matching the evidence for behavioural recovery, in the scans conducted 8–12 weeks after the lesion (late post-lesion-1), functional connectivity within the network of interest returned to the pre-lesion state (Fig. 4). The only connection that was significantly different from pre-lesion levels was a decreased correlation between left frontal and right parietal regions.

### 2.2 Changes following subsequent lesion to opposite hemisphere

Approximately seven months following the lesion to the left PS, both monkeys received a second lesion to the right PS (Fig. 2). We used a second ANOVA to evaluate the behavioural impact of this procedure across the three stages of behavioural testing: the 8-week period immediately before the second lesion (pre-lesion), the first 8 weeks following the second lesion (early post-lesion-2), and the period 8–12 weeks following the second lesion (late post-lesion-2).

The behavioural data following the second lesion are shown in Fig. 3B. Once again, we observed a significant main effect of experimental stage (*F*_(2,12)_ = 23.4, p = 0.0001) and monkey (*F*_(1,12)_ = 10.32, p = 0.0075). We also observed a significant main effect of task (*F*_(1,12)_ = 273.32, p < 0.0001). We did not observe any significant interaction between session and task (*F*_(2,12)_ = 0.24, p = 0.79). Post-hoc comparisons revealed a significant difference between the pre-lesion and both the early (p = 0.0001) and late post-lesion (p = 0.0056) periods. Though recovery was weak, we also observed a significant difference between the early- vs. late-post lesion periods (p = 0.0315).

We were unable to collect imaging data immediately prior to the second lesion. Therefore, to assess changes in connectivity associated with the second lesion, we compared functional connectivity from the scans performed after the second lesion to the latest scans after the first lesion (Fig. 1B). Thus, rsFMRI data we have referred to as late post-lesion-1 is identical to what we now refer to as pre-lesion-2.

**Fig. 5:**
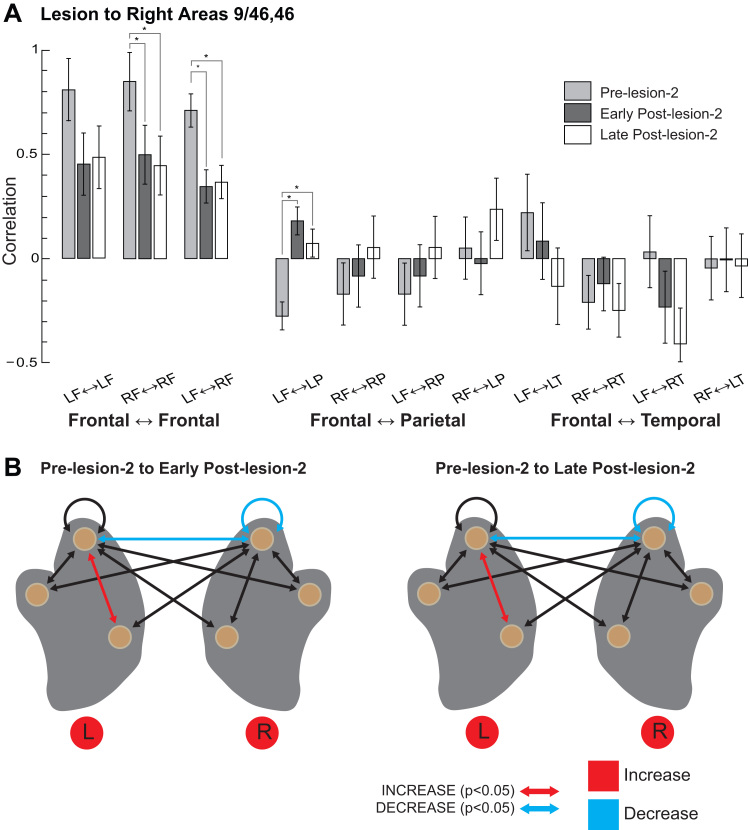
**Connectivity within frontal lobes impaired following lesion to regions near the right principal sulcus.** A) Average correlation for all frontal-frontal, frontal-parietal, and frontal-temporal connections in the pre-lesion, early-post-lesion (4–8 weeks post lesion) and late post-lesion (8–16 weeks) periods. B) Schematic representations of changes to connectivity in the early post-lesion (left) and late post-lesion (right) periods, relative to the pre-lesion period. Significant increases in functional connection strength (correlation) shown in red; decreases in blue. Filled red circles indicate lesioned hemispheres.

As before, we analysed changes in connectivity using an ANOVA (see MATERIALS AND METHODS). Overall changes in connection strength between hemispheres and lobes are shown in Fig. 5. Unlike the first lesion, the main effect for experimental stage failed to achieve statistical significance (*F*_(2,6)_ = 2.154, p = 0.20). We did, however, observe a significant main effect for connection (*F*_(10,60)_ = 20.072, p = 4.21 × 10^−7^) and a significant interaction between experimental stage and connection (*F*_(20,60)_ = 2.78, p = 0.028). We did not observe a main effect of monkey (*F*_(1,6)_ = 2.353, p = 0.18). A similar analysis looking across the whole brain revealed no significant effects of experimental stage (*F*_(2,6)_ = 0.35, p = 0.71) or monkey (*F*_(1,6)_ = 1.58, p = 0.25); and no significant interaction between stage and connection (*F*_(40,120)_ = 0.67, p = 0.93) (see Supplementary Fig. 1B).

As with the lesion to the left PS, removing the right PS led to decreased correlations within and between the frontal lobes in the early post-lesion-2 as compared to the pre-lesion-2 periods (Fig. 5). This was significant for the right hemisphere (pre-lesion-2 vs. early post-lesion-2, 0.80 ± 0.15 vs. 0.37 ± 0.15, p = 0.019), and for the inter-hemispheric correlations (0.72 ± 0.08 vs. 0.35 ± 0.08, p = 0.005). However, it did not reach statistical significance for correlations within the left hemisphere (0.74 ± 0.15 vs. 0.34 ± 0.15, p = 0.0603). We observed a significant increase in average correlations between the left frontal and parietal lobes (pre-lesion-2 vs. early post-lesion-2, 0.27 ± 0.07 vs. 0.18 ± 0.07, p = 0.019).

Compared to the first lesion, the most striking feature of network reorganisation following the second lesion was the absence of recovery in the late post-lesion period (compare Fig. 5 vs. Fig. 4). Correlations showed few differences in the late post-lesion-2 period as compared to the early post-lesion-2 period: the average right hemisphere and inter-hemispheric correlations remained significantly reduced compared to the pre-lesion levels and the correlations between left frontal and left parietal lobes remained increased relative to pre-lesion levels (pre-lesion-2 vs. late post-lesion-2, − 0.28 ± 0.07 vs. 0.07 ± 0.07, p = 0.017). Thus, weak behavioural recovery was matched by failure in recovery of frontal lobe connectivity.

## 3 Discussion

We have examined changes in functional connectivity between regions in frontal, temporal, and parietal cortex following unilateral and bilateral frontal lesions, and compared this with changes in behaviour. Following a unilateral lesion to the left PS, monkeys showed a significant impairment in performance on the DMS tasks. This was associated with a generalised decrease in connectivity between frontal regions, both within and between hemispheres. After about 8 weeks, monkeys showed an improvement in behavioural performance that was associated with largely restored connectivity within frontal regions. Following a second lesion to the opposite hemisphere, monkeys once again showed reduced connectivity within frontal-frontal connections, with some decline in behavioural performance. Importantly, following the second lesion, there was only weak recovery in behaviour and none in network integrity.

On the one hand, the network effects of a focal frontal lesion were surprisingly widespread. Following a unilateral lesion, disruption extended not just to connections between undamaged frontal regions within the lesioned hemisphere, but to connections of these to the opposite frontal lobe, and even to connections exclusively within the undamaged hemisphere. On the other hand, disrupted connectivity was far from universal. In both hemispheres, connections of frontal to parietal and frontal to temporal cortex were largely unchanged, following either unilateral or bilateral frontal lesion. Lesions also left connectivity within and between occipital, parietal and temporal lobes largely unchanged. These results suggest that, following focal frontal lobe lesions, behavioural impairments and recovery could be specifically related to a widespread disruption of connectivity; restricted to the frontal lobes but widespread within those lobes. Similarly, recovery of behaviour following a unilateral lesion was associated with bilateral recovery of frontal connectivity.

In the following sections, we first acknowledge potential limitations of this preliminary study. We then speculate why a unilateral lesion to the PS might lead to disruptions in behaviour. Next, we consider how recovery of connections within and between the frontal lobes could help compensate for the effect of the lesion and thus lead to an improvement in behavioural performance. Finally, we discuss the modest behavioural impairment and lack of network recovery following a second lesion to the opposite hemisphere.

### 3.1 Limitations and issues of interpretation

We acknowledge several limitations of this preliminary study. First, we only tested two animals, both with PS lesions. No animals without lesions or sham-operated controls were included. When designing studies in non-human primates, one must balance the need for adequate sample size with the ethical, logistical, and financial costs associated with the work. We did not anticipate a need for control animals because our experimental design used exclusively within-subjects comparisons and both animals were trained to the same criterion prior to the first lesion. In the absence of any lesion, there was no reason to suspect that the animals would exhibit significant declines in behaviour. We do not know to what degree our general findings might extrapolate to other lesion sites, nor can we directly comment on the effect of surgical manipulation alone on functional connectivity. Nevertheless, the specificity of the connectivity changes we observed does suggest the importance of our specific lesion location within the frontal lobe.

Second, we cannot be sure that underlying white matter and/or neighbouring regions were not significantly affected during the surgical procedures even though an operating microscope was used. And indeed, there is evidence from a few sections that the lesion may have extended beyond the fundus of the sulcus (Fig. 2). However, the purpose of our study was not to determine the function of the cortical tissue near the principal sulcus, but rather to study functional recovery; and in stroke patients, white matter is always affected. Regardless of the extent to which underlying white matter was affected, we were nonetheless able to observe both behavioural impairment and recovery that correlated with changes in functional connectivity.

Third, it is possible that our evidence for behavioural recovery in part reflected continuing practice effects across successive cycles of experience in the tasks. Against this, it is worth noting that we observed no improvements across successive testing days within each cycle, only between cycles, and that between cycles, animals received no further experience in the task.

Fourth, after the second lesion there were changes in functional connectivity; yet there was only a small change in behaviour. One possible confound is a practice effect, in that between the two lesions, the monkeys received more and more practice on the two tasks. However, it is important to note that both monkeys were trained over the course of several months to reach criterion prior to the first lesion and they were tested relatively infrequently following the lesion. It is therefore unlikely that practice alone can account for the behavioural recovery following the first lesion and the modest effect on behaviour of the second lesion. Another possibility is that the monkeys adopted a different strategy for completing the task that allowed them to somewhat bypass the effects of the lesions (see below).

Fifth, we collapsed the behavioural data across subject, task, testing cycles, and difficulty (i.e., delays); which raises some issues with interpretation. Given that results were broadly similar across tasks, we chose to collapse these data in order to increase statistical power and to better align the behavioural data with the imaging data. A weakness, however, is the lack of a control task with which to compare the delayed tasks. Without such a control task, it is impossible to assess whether the deficit observed is specific to delayed-matching tasks (i.e., a deficit in working memory) or something more general. Future studies would be well served by including additional tasks, including tasks where no significant behavioural impairment is expected.

Finally, we acknowledge a potential issue of voxel size. The problem is that neighbouring voxels may be supplied by the same vessels. This means that there may be an artefactual correlation between adjacent regions. To best avoid this problem, we chose as seed areas regions that were less likely to be supplied in this way (Fig. 1).

### 3.2 Why was behaviour impaired following the first lesion?

The first lesion included areas 46v (ventral bank of the PS) and 46d (dorsal bank). Despite their anatomical proximity, these two regions have quite different sets of connections, particularly with areas outside of the frontal cortex (Gerbella et al., 2013, Petrides and Pandya, 1984, Saleem et al., 2014, Yeterian et al., 2012) (Fig. 1B). Area 46d has connections to frontal areas 8 and 9; strong connections to parietal area 7, cingulate areas 23, 24 and 31; and relatively strong connections to the superior temporal gyrus (STG) and the dorsal banks of superior temporal sulcus (STS). Area 46v has connections to the ventrolateral prefrontal cortex (areas 12 and 45) and strong connections to the dorsal insula, the ventral bank of the STS, and the parietal area 7b.

On the basis of anatomical and pre-lesion functional connectivity, we speculate that there are two networks that are relevant for performance on our tasks. The first is a ‘dorsal network’, comprised of the DLPFC including areas 8, 9, and 46d, and this has extensive connections with parietal cortex and is involved in spatial encoding and, thus, in the performance of the location-based task on the basis of working memory. The second is a ‘ventral network’, comprised of the VLPFC including areas 12, 45, 46v, and 47, and this has extensive connections with the temporal cortex and is involved in object processing (Passingham and Wise, 2012), and, thus, in the performance of the object-based task. Thus, the PS lies at the anatomical intersection between these two functional networks and is in an ideal position to integrate information about location and object identity. That it does indeed do so is indicated by single-unit recording studies that have shown that neurons within the principal sulcus can have both ‘what’ and ‘where’ properties (Hoshi et al., 1998, Rao et al., 1997).

Building on this, when the tissue in both banks of the PS is damaged, we should perhaps not be surprised to see impairment in both spatially-based and object-based tasks. Our results further suggest, however, that behavioural impairments might not be explained by the damage to area 46 alone. Instead, this damage appears to broadly disrupt frontal lobe function, as suggested by the widespread reductions in frontal connectivity. Such widespread disruptions may arise precisely because area 46 itself is so broadly connected to other frontal regions, including both dorsal and ventral networks.

### 3.3 How did recovery of function occur following the first lesion and why was there only a modest deficit despite a persistent disruption to network connectivity following the second lesion?

Both monkeys eventually showed recovery of function after the first lesion, and this raises the issue of how this recovery occurs. While rsFMRI is an indirect measure of network reorganisation, we nonetheless offer the following possible explanations, which can hopefully serve as the objectives for future study.

First, we must consider the possibility that the lesions were not complete, and it was residual tissue that was mediating behavioural recovery. However, as can be seen from Fig. 2, there was very little, if any, tissue left in the principal sulcus. It is one advantage of working with animals that it is possible to ensure, as here, that the lesions are indeed complete.

A second possibility is that after the first unilateral lesion, the homotopic region in the other hemisphere could compensate. But if area 46 in the right hemisphere had indeed taken over, we might have expected to see a severe impairment when it was then itself lesioned, which was not the case (Fig. 3B); so, at best this may only be part of the answer. A third possibility, and one that is most strongly supported by the data, is that widespread cortical recovery and reorganisation concentrated within the frontal lobes contributed to the recovery of function following the first lesion. Given the complexity of the cortical network and the degree of interconnectivity, particularly within and between the frontal lobes, there are always potential alternative routes of spatial and object-based information transmission so long as the lesion is not extensive. If there are multiple potential strategies to complete the task, for example, it is conceivable that alternative pathways that bypass area 46 might be recruited, or might recover, as frontal connectivity is restored.

One possibility is that on the spatial task the monkeys were able to adopt an attentional strategy. There are many cells in the ventral prefrontal area 45 that respond when monkeys attend to a stimulus (Lebedev et al., 2004) and a possible strategy is that the monkey could attend to the relevant location on the spatial task throughout the delay. There are outputs from this region to the preSMA and dorsal premotor cortex via the dorsal prefrontal areas 8 and 9 (Takahara et al., 2012). To directly test this hypothesis, we might record neuronal activity from DLPFC both pre- and post-lesion, and/or inactivate (through permanent lesion or temporary approach) DLPFC in these animals and observe its impact on behaviour. Thus, comparing pre- and post-lesion functional connectivity networks can perhaps not directly answer the question of what leads to recovery, but it can highlight potential hypotheses and opportunities for further study.

Results following the second lesion were somewhat mixed. The behavioural impairment was modest in comparison to the large impairment that followed the first unilateral lesion, and recovery even more modest. The disruption of frontal lobe connectivity was again substantial and this time long-lasting or permanent. Potentially, behaviour is only mildly affected by a second lesion to the right area 46 because by this point, the animals have either adopted a strategy that is less dependent on area 46 and/or alternative neural pathways that bypass area 46 have been recruited. Moreover, assuming this is true, there would be little drive for cortical reorganisation in this case, which would account for why little change in network connectivity was observed even at the late post-lesion-2 period. A second possibility is that, for frontal networks to recover, area 46 is necessary in at least one hemisphere, relating to the common clinical observation that especially severe cognitive deficits can follow bilateral frontal lesions. In this case, modest behavioural impairment following the second lesion might be ascribed to some additional factor, such as the additional training received between the two lesions.

## 4 Summary and future applications

In line with many previous suggestions, our data suggest that the effects of a focal frontal lobe lesion cannot be understood simply as loss of function in the specific area removed. Instead, there appears to be widespread, though also specific, disruption of connectivity between many regions within and between the two frontal lobes, potentially bringing a widespread impairment in their function. At least following a unilateral lesion, this disruption recovers over time and can be associated with recovery in behaviour as well. We suggest that it is only possible to understand how a brain lesion affects behaviour by studying the whole network and its interconnections. A lesion, however discrete, disturbs the network as a whole and this effect can last for weeks. However, recovery can occur as intact parts of the network regain their normal function, providing alternative ways in which the system can perform the task. Given the complexity of the network, there are multiple ways in which information in one area can be transmitted to inform another.

With this study, we contribute to the development of a framework for investigating recovery after lesions that relies on a non-invasive methodology (rsFMRI). Critically, this methodology allows for longitudinal tracking of cortical reorganisation. Above, we have highlighted one potential application for data such as these (identifying potential targets for inactivation in order to test mechanisms of recovery). Given enough data, this methodology could be used to generate predictions about clinical outcomes based on markers of cortical reorganisation following stroke, as in the study by Siegel et al. (2016). The use of an animal model such as ours could be used to develop new therapies for promoting recovery in patients by driving cortical reorganisation. For example, cortico-cortical paired associative stimulation (ccPAS) has been used to enhance functional connectivity between visual areas leading to increased visual perception (Romei et al., 2016) and motor plasticity (Chao et al., 2015, Johnen et al., 2015). However, fully evaluating the potential of techniques such as paired stimulation in driving *targeted* post-lesion recovery requires assessment of baseline connectivity prior to and post lesion. Such an assessment is feasible, as in our study, in a non-human primate model.

## Appendix A Supplementary material

Supplementary material **Supplementary Fig. 1 Connectivity outside frontal network following lesion to regions near left (A) and right (B) principal sulcus.** Average correlation for pairwise connections between parietal, temporal, occipital regions in the pre-lesion, early-post-lesion (4–8 weeks post lesion) and late post-lesion (8–16 weeks) periods for the first (A) and second (B) lesions. No significant interaction between stage and connection was observed in either case (p’s > 0.05).
