# Supplementary material for: Functional reorganisation and recovery following cortical lesions: A preliminary study in macaque monkeys
Source: Neuropsychologia. 2018 Oct;119:382–91. doi: 10.1016/j.neuropsychologia.2018.08.024 (PMC6200854; doi:10.1016/j.neuropsychologia.2018.08.024)
Supplement: Supplementary file 1 — Supplementary material Supplementary Fig. 1 Connectivity outside frontal network following lesion to regions near left (A) and right (B) principal sulcus. Average correlation for pairwise connections between parietal, temporal, occipital regions in the pre-lesion, early-post-lesion (4–8 weeks post lesion) and late post-lesion (8–16 weeks) periods for the first (A) and second (B) lesions. No significant interaction between stage and connection was observed in either case (p’s > 0.05). [file mmc1.docx]

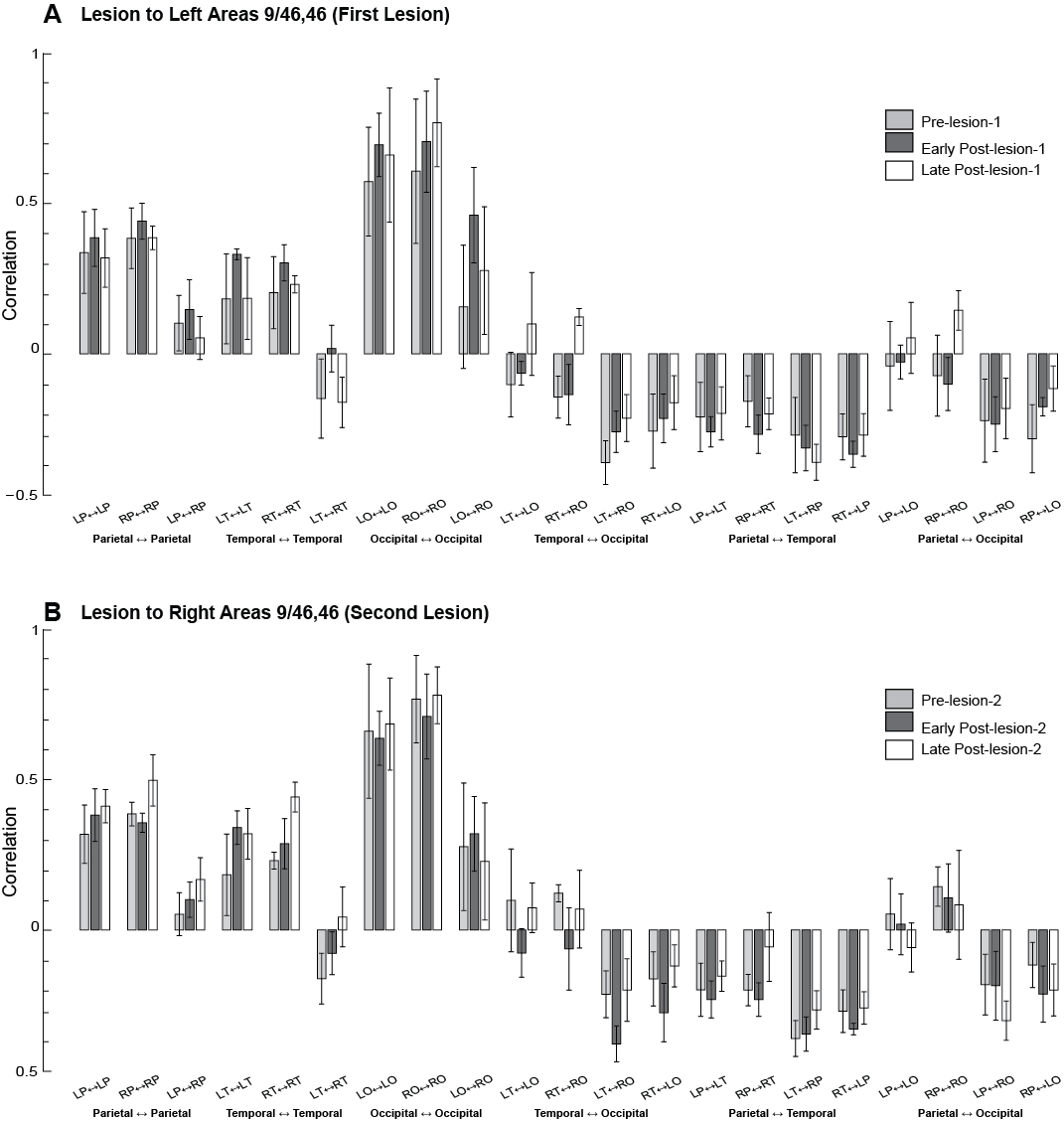


**Supplementary Figure 1 – Connectivity outside frontal network following lesion to regions near left (A) and right (B) principal sulcus.** Average correlation for pairwise connections between parietal, temporal, occipital regions in the pre-lesion, early-post-lesion (4-8 weeks post lesion) and late post-lesion (8-16 weeks) periods for the first (A) and second (B) lesions. No significant interaction between stage and connection was observed in either case (p’s>0.05).
